# The effect of acupuncture duration on analgesia and peripheral sensory thresholds

**DOI:** 10.1186/1472-6882-8-18

**Published:** 2008-05-01

**Authors:** Albert Y Leung, Susan J Kim, Gery Schulteis, Tony Yaksh

**Affiliations:** 1 Department of Anesthesiology, The University of California, San Diego, School of Medicine, La Jolla, CA, USA; 2 VA San Diego Healthcare System, La Jolla, CA, USA; 3 The University of California, San Diego, School of Medicine, La Jolla, CA, USA

## Abstract

**Background:**

Acupuncture provides a means of peripheral stimulation for pain relief. However, the detailed neuronal mechanisms by which acupuncture relieves pain are still poorly understood and information regarding optimal treatment settings is still inadequate. Previous studies with a short burst of unilateral electroacupuncture (EA) in the Tendinomuscular Meridians (TMM) treatment model for pain demonstrated a transient dermatomally correlated bilateral analgesic effect with corresponding peripheral modality-specific sensory threshold alterations. However, the impact of EA duration on the analgesic effect in this particular treatment model is unknown. To obtain mechanistically and clinically important information regarding EA analgesia, this current prospective cross-over study assesses the effects of EA duration on analgesia and thermal sensory thresholds in the TMM treatment model.

**Methods:**

Baseline peripheral sensory thresholds were measured at pre-marked testing sites along the medial aspects (liver and spleen meridians) of bilateral lower extremities. A 5-second hot pain stimulation was delivered to the testing sites and the corresponding pain Visual Analog Scale (VAS) scores were recorded. Three different EA (5Hz) stimulation durations (5, 15 and 30 minutes) were randomly tested at least one week apart. At the last 10 seconds of each EA session, 5 seconds of subject specific HP stimulation was delivered to the testing sites. The corresponding pain and EA VAS scores of de qi sensation (tingling) during and after the EA were recorded. The measurements were repeated immediately, 30 and 60 minutes after the EA stimulation. A four-factor repeat measures ANOVA was used to assess the effect of stimulation duration, time, location (thigh vs. calf) and side (ipsilateral vs. contralateral) of EA on sensory thresholds and HP VAS scores.

**Results:**

A significant (P < 0.01) main effect of time and location with warm, cold and hot pain thresholds at the four testing sites without any significant difference in duration effect was observed. Similar time and location effects were observed with HP VAS with the longer durations (15 and 30 minutes) of stimulation showed a slower onset, but a more sustainable bilateral analgesic benefit than the short stimulation duration (5 minutes). The 15-minute stimulation resulted in an earlier onset of analgesic effect than the 30-minute stimulation paradigm.

**Conclusion:**

Longer durations of EA stimulation provide a more sustainable analgesic benefit to hot noxious stimulation than a shorter duration of stimulation. The increase of cold threshold with sustained warm threshold temperature elevation as observed in the longer durations of EA suggests that as the duration of EA lengthened, there is a gradual shifting from an initial predominantly spinally mediated analgesic effect to a supraspinally mediated modulatory mechanism of thermal pain. The 15-minute stimulation appeared to be the optimal setting for treating acute pain in the lower extremities.

## Background

Despite recent studies which indicate that acupuncture is effective in treating chronic pain, the mechanisms by which acupuncture relieves pain are poorly understood and information regarding optimal treatment settings is still inadequate [1-6]. With quantitative peripheral sensory testing and functional magnetic resonance imaging (fMRI) as assessment tool, the Tendinomuscular Meridian (TMM) system which empirically used for treating acute pain was recently adopted as a means to uncover some of the key scientific basis of acupuncture analgesia [7-11]. A previous study with unilateral Ting Points (TPs) electroacupuncture (EA) of the lower extremities within the TMM system demonstrated a transient dermatomally correlated analgesic effect at the bilateral calf areas, but not at the thigh areas [8]. A follow-up study with additional needling locations as described in the original treatment protocol provided an expanded dermatomally correlated analgesic effect at the thigh areas [9]. The punctate nature of EA with the elevation of warm thresholds at the bilateral calf areas suggests an A-delta afferent fiber mediated C-fiber modulation [8]. In addition, the bilateral peripheral analgesic effect with a dermatomal correlation further supports this notion that a short burst of EA at the TPs can facilitate centrally mediated nociceptive inhibitory effect. However, to our best knowledge, no studies have been done to adequately assess the effect of acupuncture duration on analgesic effect using this particular model. Therefore, in the current study, the authors expanded their investigation by assessing the effect of stimulation duration on acupuncture neuronal analgesic mechanisms via peripheral sensory thresholds and behavioral response assessments.

## Methods

With the University of California, San Diego, human subject review committee (IRB) approval, 16 healthy subjects (10 females and 6 males) were recruited for the study based on the study's inclusion and exclusion criteria as shown in table 1.

**Table 1:** Subjects' inclusion and exclusion criteria

Inclusion criteria	Exclusion criteria
Age 18 to 80	History of psychological illness
Male or Female	History of claustrophobia
No analgesics for the past 2 weeks	Pregnancy
Absence of neuropathic pain states	Pending litigation
No acupuncture treatment for the past 2 weeks	History of head trauma
	History of trauma or surgery to lower extremities and pelvic area

**Figure 1:**
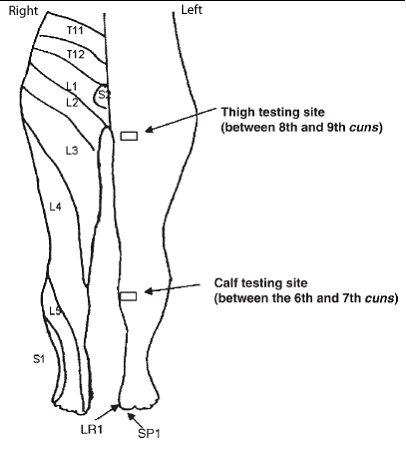
Dermatomal Relationship for EA and Testing Sites.

Baseline thermal thresholds (cold, warm, cold pain, and hot pain) and von Frey (VF) tactile thresholds of bilateral medial calves (between 6^th ^and 7^th ^cun) and thighs (between 8^th ^and 9^th ^cun) were measured at pre-marked testing sites via a Peltier Thermal Analyzer (Medoc Advanced Medical Systems, Durham, NC). All study sessions were conducted in a study room where the thermostat of the room was set at 20°C. The location of needle placement, the testing sites, and their corresponding dermatomal relationship is shown in Figure 1. Five seconds of hot pain (HP) stimulation at individually determined HP thresholds were delivered to the subject's testing sites and the corresponding pain visual analog scale (VAS) scores were recorded.

Acupuncture needles were placed at the following locations:

1) SP1 (Yinbai) – on the dorsal aspect of the great toe, at the junction of lines drawn along the medial border of the nail and the base of the nail, approximately 0.1 cun from the corner of the nail [12]

2) LR 1 (Big Mound) – on the dorsal aspect of the great toe, at the junction of lines drawn between the lateral border of the nail and the base of the nail, approximately 0.1 cun from the corner of the nail [12]

In a randomized fashion, a 3-session series (five-, fifteen-, and thirty-minute) EA was delivered at the TPs at 5 Hz with a pulse duration of 300 μs and an intensity of 8 out of a maximum of 10 on a 6V square-wave stimulator (Eto-160, Japan) at least one week apart. During the last 10 seconds of each EA session, 5 seconds of subject-specific HP stimulation at the pre-EA threshold were delivered to the left calf testing site. The VAS scores of *de qi *sensation (tingling) throughout EA and the HP VAS score at the end of EA were then recorded. Baseline thermal threshold and HP VAS measurements were repeated at each testing site immediately (Post-0), as well as 30 minutes (Post-30) and 60 minutes (Post-60) after each EA session.

### Acupuncture

One-inch long 36 G gold-plated sterile acupuncture needles were used for the study because of its fMRI compatibility for later correlation studies. An experienced medical acupuncturist performed all the needle insertions in all the subjects. The needles were pre-marked sterilely by a surgical marking pen so that the depth of needle placement was between 0.5 and 1 cm.

### Cun Measurement and Testing Sites

Testing sites were chosen along the SP and LR meridians at the medial aspect of the calves and thighs. In the classical acupuncture literature, the distance of an acupuncture point from a certain anatomical landmark was measured by a unit called cun. The number of cun between different parts of anatomical landmarks is well established in acupuncture literature. Given the difference in body lengths and sizes of the subjects and for consistency in the study, an elastic ribbon that was approximately the size of a 1-inch wide ruler with marked units was used to indicate the correct number of divisions (cun) for that body region and the sites of testing and stimulation were then marked. At the medial calf, the measurement was between the 6^th ^and 7^th ^cun measuring from the medial condyle to the medial malleolus of the tibia (a total of 13 cun). At the medial thigh, the site of study was between the 8^th ^and 9^th ^cun measuring from the midline of the superior border of the pubic symphysis to the medial epicondyle of the femur (a total of 18 cun).

### Needle Placement Location

Anatomical landmarks were first used to mark the approximate locations of acupuncture needle placement and the exact locations of the needle placement were further confirmed by measuring the electroconductivity of the needle placement site at the TPs. A clinical acupuncture electroconductivity measuring device (Point Finder, Hong Kong) with a preset clinically acceptable sensitivity level of 3 out of a maximum 10 was used for located the exact location of the acupuncture needle placement. The locations were then marked with a surgical marking pen.

### *De Qi *Sensation

The *de qi *sensations are known qualitatively as having different components of sensations such as dull aching, needle grasping, heaviness, electrical-like (tingling) in different acupuncture texts [13-16]. A recent electrophysiology study showed that tingling is the most common sensation felt with EA[17]. Therefore, in the current study, the subjects were primarily asked to rate their *de qi *sensation based on the degree of tingling that they felt on a linear VAS.

### Peripheral Sensory Testing

Non-noxious thermal sensations including cold and warm, and noxious thermal sensations such as cold pain and hot pain thresholds were measured by using a Thermal Sensory Analyzer (Medoc Advanced Medical Systems, Durham, NC, USA). This device consisted of a thermode measuring 46 × 29 mm. The temperature of the thermode could either rise or fall (at a rate of 1.2 degrees Celsius/sec for cold and warm sensations, and 3 degrees Celsius/sec for cold and hot pain), depending on the sensations that were being tested. The subject signaled the onset of feeling the tested sensation by pressing a switch, which in turn reversed the temperature change and returned the temperature of the thermode to the 32 degree Celsius baseline temperature. The computer then recorded the temperature of the thermode when the switch was pressed. The average value of the testing results was automatically calculated by the computer and displaced to the screen. This method of peripheral sensory testing has been well established in the literature and has been used extensively in pain-related studies [18,19]. The tactile sensation was measured by using the von Frey (VF) hair monofilaments of varying size(North Coast Medical, Morgan Hill, CA, USA). The testing was done in a descending fashion. Each filament was tested three times. Each test lasted about five seconds with a ten-second break in between. The tactile threshold was determined if the patient felt at least two of the three consecutive testing measurements and none from the next smaller-sized filament.

### Visual Analog Scale and Behavioral Measurement

The visual analog scale (VAS) is a horizontal linear scaled with a length of 100 mm. One end of the scale was marked "No Pain" or "No Tingling" while the other end of the scale was marked "Worst Pain Imaginable" or "Maximum Degree of Tingling". These two different sets of marking were used for the HP and *de qi *sensations, respectively.

### Data Analysis

Data on sensory thresholds and behavioral response to hot pain were entered into a four-factor repeated measures ANOVA (analysis of variance), with duration of EA stimulation (five, fifteen, vs. thirty minutes of stimulation), time-course (pre- and post-stimulation), location of measurement (calf vs. thigh), and side of the body (ipsilateral vs. contralateral) as the factors. A two-tailed p-value of less than 0.05 was considered significant. In some cases further exploration of significant interactions were completed by post-hoc individual means comparisons using the Bonferroni correction to maintain an overall (family wise) error rate of p < 0.05.

## Results

With the University of California, San Diego Human Subject Review Committee approval and written informed consent, 16 subjects (10 females and 6 males) were enrolled for the study. The median age for this cohort was 25.5 years, with an age range of 19 to 49 years. With only 6 male subjects enrolled, this study was not specifically powered to detect gender differences, but an exploratory analysis attempted to identify possible differences between males and females in either a) baseline thresholds and VAS ratings or b) response to stimulation. This analysis revealed no reliable gender differences, and accordingly subjects were pooled for all subsequent statistical analyses.

### 1) Cold Sensation Threshold

**Figure 2:**
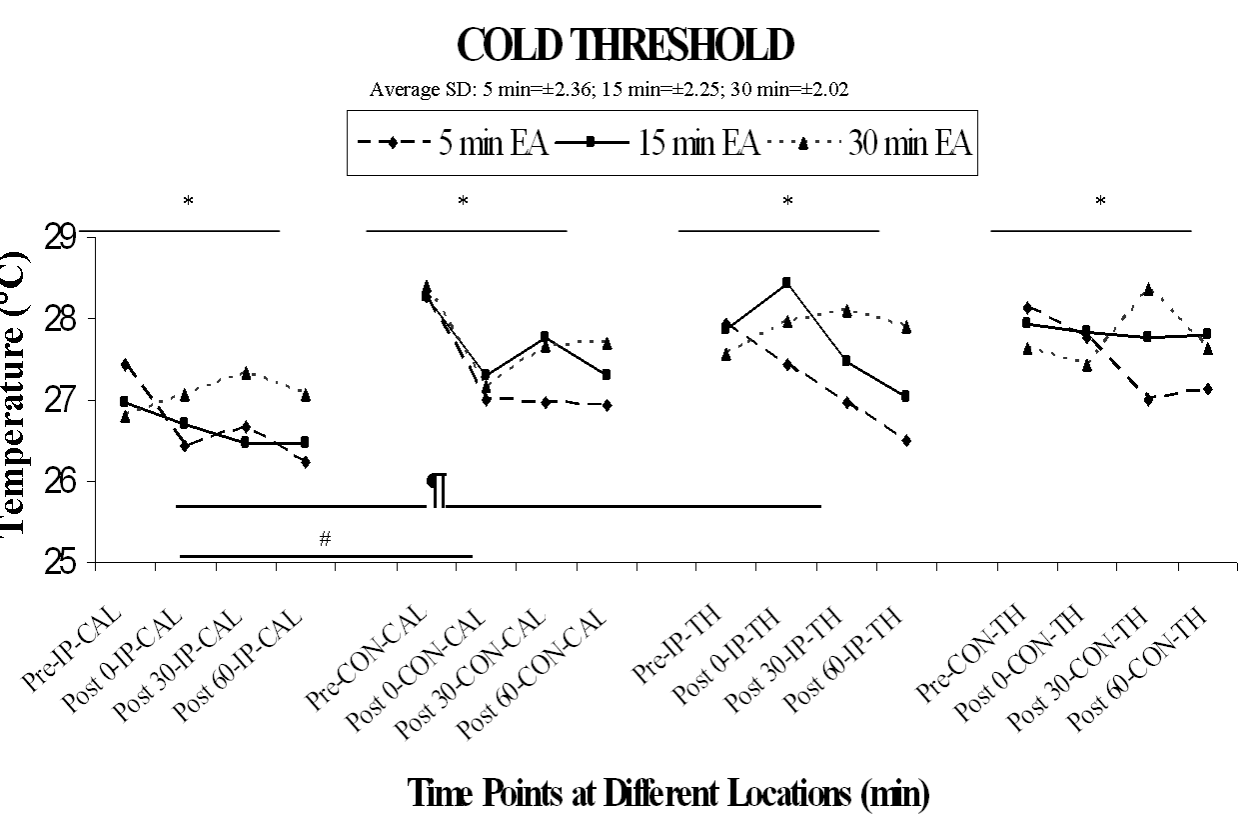
**Cold threshold**. The four-factor repeated measures ANOVA revealed a significant main effect of location ¶(F [1, 15] = 15.4, p < 0.01), main effect of time* (F [3, 45] = 3.1, p < 0.05), and main effect of side# (F [1, 15] = 5.7, p < 0.05), with the ipsilateral calf threshold temperatures being mostly lower than the ipsilateral thigh threshold temperatures.

Results are illustrated in Figure 2. The overall four-factor repeated measures ANOVA revealed a significant main effect of location (F [1,15] = 15.4, p < 0.01), main effect of time (F [3, 45] = 3.1, p < 0.05), and main effect of side (F [1,15] = 5.7, p < 0.05), and a location × side interaction that approached significance (F[1,15]= 4.27, p = 0.0565). These significant effects can be accounted for by lower cold thresholds at the ipsilateral calf location than at the ipsilateral thigh, contralateral calf, or contralateral thigh, but this effect was evident pre-stimulation and therefore not a direct result of stimulation (see Figure 2). With regard to the primary variable of interest in the current study, however, there was no significant main effect of duration, but there was an interaction of duration and time (F [6,90] = 2.23, p < 0.05), with the longest duration of stimulation (30 min) producing less change from pre-stimulation baseline than the shortest duration (5 min); the remaining interactions with the duration factor did not achieve significance (all F's < 1.41, P's > 0.20).

### 2) Warm Sensation Threshold

**Figure 3:**
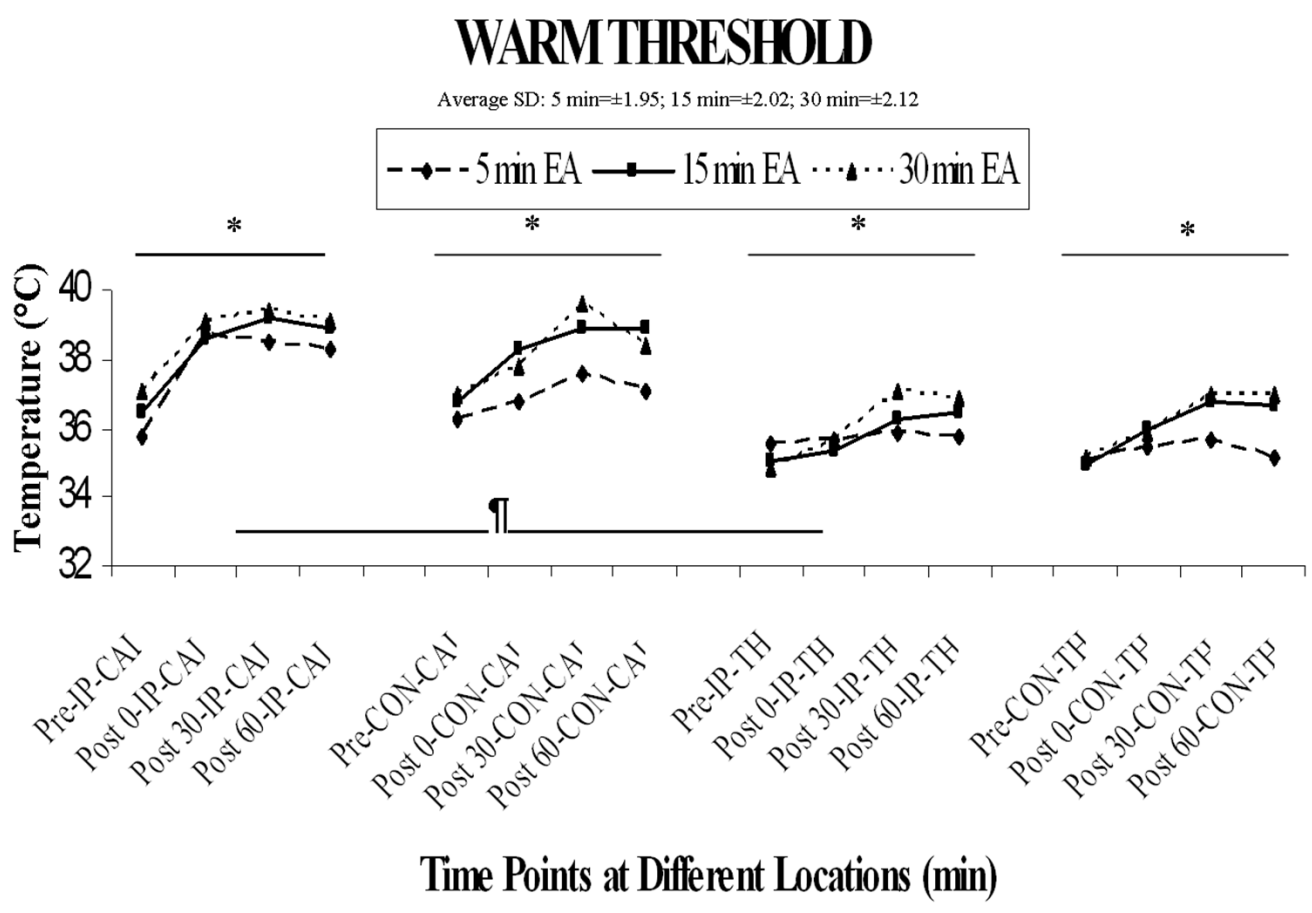
**Warm threshold**. The overall four-factor repeated measures ANOVA revealed a significant main effect of location ¶(F [1, 15] = 82.9, p < 0.0001), significant main effect of time* (F [3, 45] = 35.7, p < 0.0001), and a significant interaction of location and time (F [3, 45] = 8.0, p < 0.001). The main effect of location was accounted for by the significantly higher thresholds in calf versus thigh at all time points.

The overall four-factor repeated measures ANOVA revealed a significant main effect of location (F [1, 15] = 82.9, p < 0.0001), significant main effect of time (F [3, 45] = 35.7, p < 0.0001), and significant interactions of location and time (F [3, 45] = 8.0, p < 0.001) as well as location × side × time (F [3,45] = 4.89, p < 0.005). The main effect of location can be accounted for by a significantly higher threshold in calf versus thigh at all locations and time points including pre-stimulation (see Figure 3). The significant interactions with the location factor are accounted for by proportionately greater increases in warm threshold from pre- to post-stimulation in the calf vs. thigh region. With regard to the primary factor of interest in the current study, however, there was no significant main effect of duration or interaction of duration with any other factor (all F's < 2.85, P's > 0.05), suggesting that duration of stimulation does not significantly influence warm sensation thresholds.

### 3) Cold Pain Threshold

As noted in previous studies, large variance of cold pain threshold temperatures was observed among subjects. A four-factor ANOVA revealed a significant main effect of duration (F [2,30] = 6.3, p < 0.005) and a significant main effect of time (F [3, 45] = 3.5, p < 0.05). The main effect of time was likely due to a modest progressive reduction in cold pain thresholds at all measurement locations and stimulation durations. The main effect of duration was due to a significantly higher cold pain threshold in the 5 min duration condition than in the 15 or 30 min duration, but this effect was present prior to stimulation (e.g. pre-stimulation baseline), and therefore cannot be attributed to the effect of stimulation per se, but rather reflects a nonspecific difference in cold pain thresholds across test days. No other significant main effects or interactions among any combination of factors were significant (all Fs < 2.0, p > 0.10).

### 4) Hot Pain Threshold

**Figure 4:**
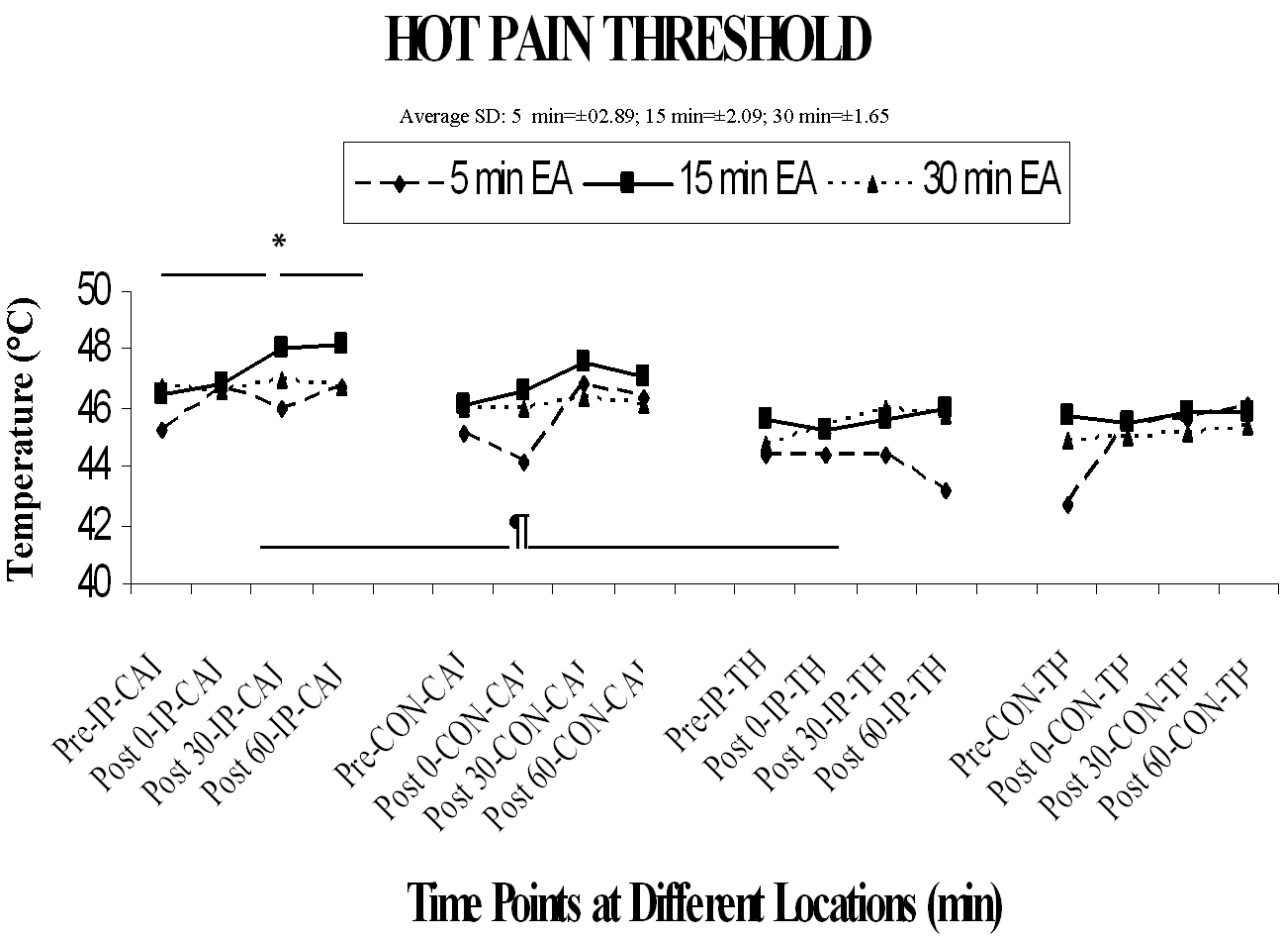
**Hot pain threshold**. The four-factor ANOVA revealed a significant main effect of location ¶(F [1, 15] = 16.8, P < 0.001) and main effect of time * (F [3, 45] = 8.5, P < 0.0001).

Overall ANOVA analysis of hot pain thresholds revealed a significant main effect of location (F[1,15] = 16.83, p < 0.0009) and a significant main effect of time (F [3,45] = 8.50, p < 0.0001)and an interaction of location × side that approached significance (F[1,15] = 3.94, p – 0.066). As shown in Figure 4, hot pain thresholds at the calf are slightly higher than at the thigh location. With regard to the primary factor of interest, the main effect of duration approached significance (F[2,30] = 3.02, p = 0.064), but there was no significant interaction of duration with any other factor (all F's < 1.92, P's > 0.05). An inspection of Figure 4 indicates that thresholds appeared to be somewhat lower on the day of 5 min duration stimulation, but as with cold pain thresholds this effect was present pre-stimulation, and therefore cannot be specifically attributed to an effect of varying duration of stimulation per se.

### 5) Hot Pain VAS scores

**Figure 5:**
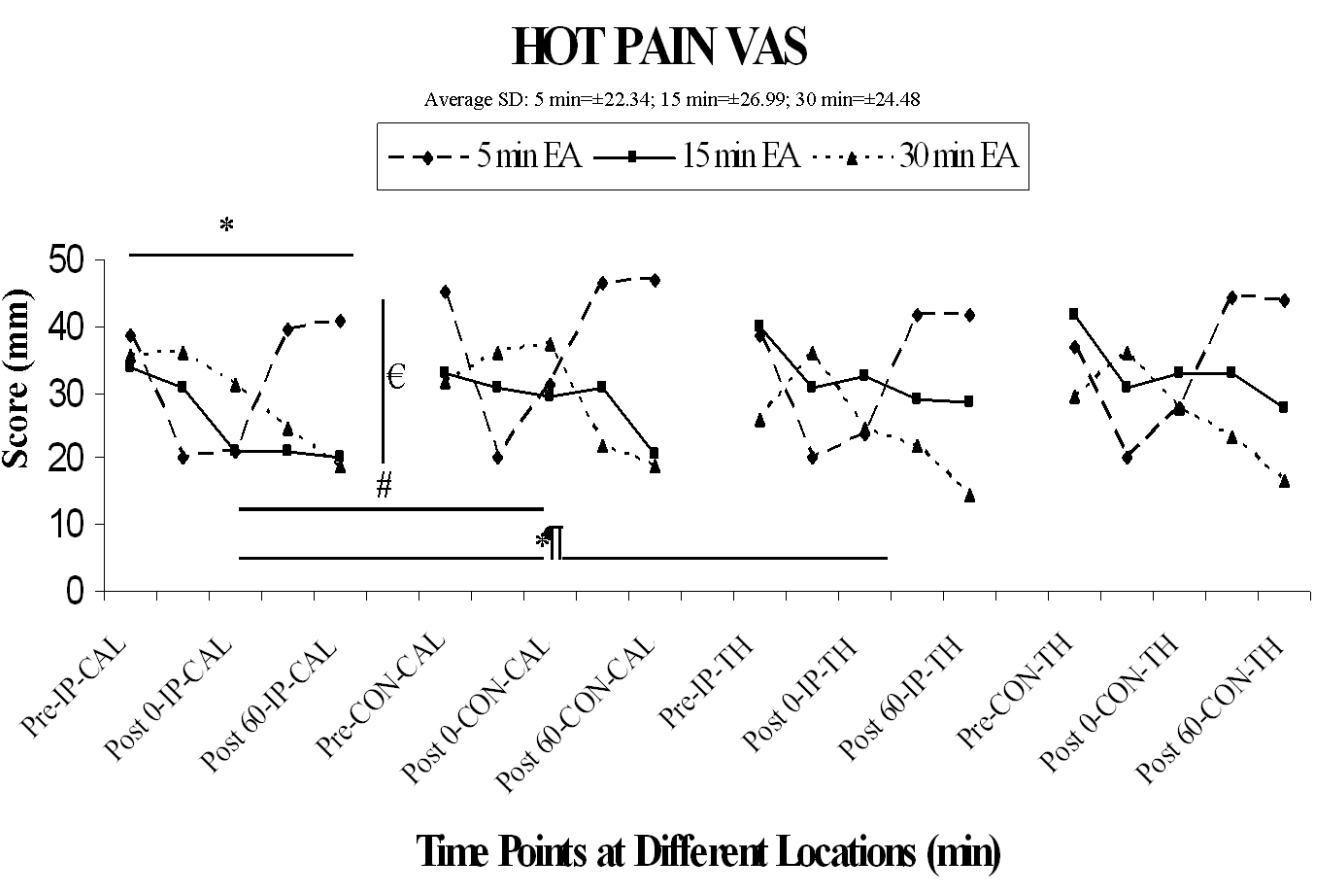
**Hot pain VAS**. There is a significant main effect of duration €(F [2, 30] = 6.1, p < 0.01), a main effect of side (F [1, 15] = 6.3, p < 0.05), and a main effect of time * (F [3, 45] = 3.5, p < 0.05). In addition, a significant interaction of duration and location ¶(F [2, 30] = 6.6, p < 0.005) and of duration and time (F [6, 90] = 5.6, p < 0.0001) were noted.

In contrast to hot pain thresholds, the hot pain VAS scores appeared to vary in a duration-dependent fashion (see Figure 5). The effect of the 5-minute stimulation duration on the HP VAS appeared rather transient, whereas the effects of the 15- and 30-minute stimulation durations seemed to generate a much more sustainable decline in HP VAS. These observations were confirmed by a four-factor ANOVA, wherein there were significant main effects of duration (F [2,30] = 6.09, p < 0.01) and time (F [3,45] = 3.49, p < 0.05), as well as significant interactions of duration with location (F [2,30] = 6.64, p < 0.005) and with time (F [6,90] = 5.65 p < 0.0001).

Since HP VAS score was obtained during last 10 seconds of each stimulation at the ipsilateral calf, an additional two factor repeated measures ANOVA with duration and time including a time point during stimulation was performed on the data from this location. Results included a significant main effect of time (F [4,60] = 3.36, p < 0.02), and a significant interaction of duration and time (F [8,120] = 4.91, p < 0.0001). The duration × time interaction was further dissected using post-hoc comparisons of each stimulation time point (during, post-0, post-30, post-60) to pre-stimulation under the different stimulation conditions, using the Bonferroni correction with the overall (family wise) error rate held constant at p < 0.05:

a) In the 5-minuted stimulation paradigm, HP VAS scores were significantly reduced during and immediately post (post-0) stimulation, but had returned to baseline values by 30 and 60 min.

b) In the 15-minute stimulation paradigm, there was no significant change during or immediately post-stimulation, but a significant reduction in HP VAS had emerged by 30 min post-stimulation, and this effect persisted to at least 60 min.

c) In the 30-minute stimulation paradigm, there was again no reduction in HP VAS score during or immediately post-stimulation, and the effect at 30 min approached, but did not achieve significance; however, there was a robust reduction in HP VAS scores at 60 min post-stimulation.

### 6) von Frey Threshold

A four-factor ANOVA revealed no significant main effects or interactions among any combination of factors (all Fs < 1.1, p > 0.10). No significant difference of baseline von Frey tactile thresholds were observed between calves and thighs and no significant changes over time were noticed at any of the four testing sites.

### 7) *De Qi *sensation

**Figure 6:**
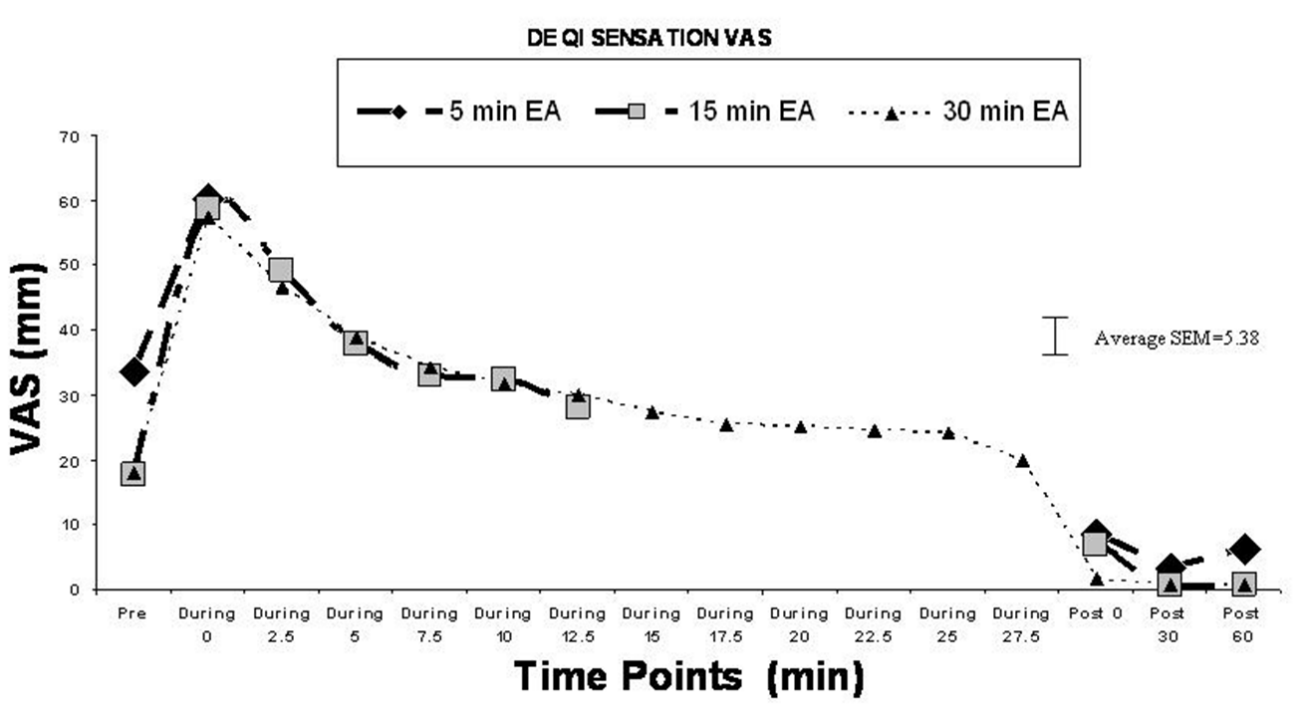
De Qi sensation and VAS score.

The degree of *de qi *sensation increased significantly at the onset of EA and decreased gradually during the EA session (see Figure 6). Four separate one-factor ANOVAs (one for each location of heat probe application) on *de qi *VAS scores revealed a time-dependent change in *de qi *VAS scores at all measurement locations (all Fs > 11.3, p < 0.001). Follow-up pair-wise comparisons confirmed that in all cases, the VAS scores increased significantly during stimulation from pre-stimulation values, and in all cases, declined back to pre-stimulation values immediately after the EA stimulation was turned off (i.e. post-stimulation).

## Discussion

### The effect of stimulation durations on analgesic efficacy

In the current study, longer durations of EA stimulation at the TPs (SP1 and LR1) around the L4 dermatome of the left lower extremity resulted in an extended area of analgesia along the medial aspect of the bilateral lower extremities in comparison to the analgesic effect observed in the short burst EA stimulation study conducted previously[8]. This assertion is supported by the fact that a significant sustainable reduction of HP VAS was observed in both the 15-minute and the 30-minute stimulation paradigms, whereas the 5-minute stimulation paradigm resulted in a very quick onset, but transient analgesic benefit that is comparable to the result observed in the previous study with a 30-second stimulation paradigm. Interestingly, in the previous study with a short burst of 30-second stimulation, the main analgesic effect observed was only limited to the bilateral calf areas correlated to the dermatomal distribution of the stimulated TPs at around L4 and L5 dermatomes, but not in the thigh areas. The extended analgesic effect with a short burst of stimulation was only achieved by adding additional needle at the gathering point (CV2) which consisted of a close dermatomal correlation to the thigh areas being tested [9]. We exclude the possibility that this observed effect is mediated through a simple spinal reflex mechanism as the analgesic effect was observed in bilateral lower extremities instead of just in the lower extremity ipsilateral to the site of EA stimulation. Interestingly, in the current study, the onset of analgesic is much more rapid with the 5-minute stimulation than the longer stimulation paradigms. However, the duration of analgesic effect of the 5-minute stimulation paradigm is much shorter than the longer stimulation paradigms. Of all three stimulation paradigms, the onset of the analgesic effect is the slowest with the 30-minute stimulation paradigm. The observed result was quite consistent with the result of studies in which longer durations of stimulation resulted in different degree of analgesic benefit in comparison to shorter duration of stimulation. Recent human studies with fMRI also suggest different durations of stimulation may result in different centrally originated analgesic mechanisms [20-22].

### Analgesic mechanisms of EA

Based on previous sensory modality-specific studies, generally speaking, high-frequency and low-threshold mechano-stimulation is transmitted by the myelinated A-beta fibers. Cool, punctuate tactile sensation and well-localized pain is carried by the smaller myelinated A-delta fibers, whereas warm, hot and cold pain sensations are largely carried by the unmyelinated C-fibers[23,24]. Previous nerve block studies also suggest that acupuncture can have a neuronal modulatory effect via segmental and/or suprasegmental mechanisms on noxious peripheral afferent inputs, largely mediated via the myelinated fibers [25-28]. In the previous study with the TPs short burst EA stimulation, only C-fiber mediated sensory (warm) threshold changes was observed, whereas change in A-delta afferent mediated sensory threshold such as von Frey and cold pain were not noticed[8]. Yet in this current study, changes in both warm (C-fiber) and cold sensation (A-delta fiber) thresholds were observed in all three stimulation duration with the increase of cold threshold temperatures and sustained warm threshold temperature elevation. We excluded this observation was due to an adaptation phenomenon as the previous adaptation paradigms with repeated sensory thresholds measurement showed no significant changes over time. Therefore, the observed non-noxious thermal thresholds elevation with subsequent diminished behavioral response to thermal pain suggests that EA stimulation at the TPs not only has a sustainable modulatory effect on the afferent C-fiber itself, but the longer duration of stimulation also affects A-delta afferent fiber-mediated sensory modalities. Furthermore, in this current study, the modulatory effect on the C-fiber as reflected via warm and hot pain threshold changes appears to be more sustainable than the modulatory effect on the A-delta fiber as reflected by cold threshold changes. Thus, longer durations of EA stimulation provided a more sustained analgesic benefit to hot noxious stimulation as demonstrated via the contrast in HP VAS scores in comparison with short durations of treatment. Since both A-delta fibers and C-fibers are afferent nociceptive fibers and the inhibitory effect of A-delta afferent on the C-fiber afferent-mediated nociception is well known, the current observation supports the notion that longer durations of EA stimulation facilitates a shifting of A-delta mediated C-fiber modulation to a supraspinally mediated modulatory mechanism of thermal pain [29-31]. This postulation was supported by an earlier observation that there was a high correlation between the tingling component of the acupuncture sensation (de qi) and peripheral electrical conductance elevation with EA, suggesting an EA induced peripheral sensory fiber activation [17]. In addition, mechanical punctate sensation as observed in acupuncture or von Frey filaments can be primarily A-delta mediated[32]. This initial peripheral A-delta mediated pain inhibition likely consists of a spinal mechanism as we have observed a dermatomally correlated bilateral analgesic effect at the calves with a short burst of unilateral TP EA stimulation [8]. In addition, this shifting from an initially peripheral afferent stimulation induced and spinally mediated pain inhibitory mechanisms to a supraspinally mediated modulatory mechanism with a long duration of stimulation was also supported by the fact that the duration of the analgesic effect outlasted the de qi sensation of the EA stimulation and the observed analgesic effect in bilateral lower extremities extended beyond the dermatomally correlated areas with the unilateral TP EA stimulation.

As observed in previous studies, the baseline sensory threshold at the testing sites appeared to be slightly different between the calf and thigh with the later having a lower warm threshold temperature than that of the former. As proposed previously, this observation suggested the thigh has a higher density of A-delta and C-fiber afferent innervations than the calf [8,9]. Given the crossover design of the study, limitation of the study was that no sham acupuncture was used. However, a pervious adaptation paradigm showed that repeated thermal sensory measurements and thermal stimulation did not have any significant effect on the outcome under similar experimental conditions [8].

### Optimal Setting for Clinical Practice

The result of this study demonstrated that by extending the duration of high intensity and low frequency stimulation at the TPs the lower extremities, one can provide a prolonged duration and extended areas of analgesic benefit at the lower extremities. However, given that the 15-minute stimulation paradigm was able to generate a more significant reduction of HP VAS scores with an earlier onset of analgesia than the 30-minute stimulation paradigm, stimulation duration near 15 minutes and less than 30 minutes will likely be the ideal treatment paradigm for acute pain in the extremities.

## Conclusion

The results of the current study suggest that longer durations of EA stimulation provide a more sustained analgesic benefit to hot noxious stimulation as demonstrated via the contrast in HP VAS scores of the different stimulation durations. In comparison to a shorter duration of EA, the increase of cold threshold temperatures with sustained warm threshold temperature elevation and the lingering analgesic effect after the cessation of EA sensation as observed in the current study suggests that the longer duration of EA can better facilitate supraspinally mediated modulatory mechanism of thermal pain. The 15-minute stimulation paradigm appeared to generate the optimal treatment setting for acute pain in the lower extremities.

## Competing interests

The authors declare that they have no competing interests.

## Authors' contributions

AYL conceived of the study, and participated in the original design, and coordination and drafted the manuscript. SJK carried out the study and helped to draft the manuscript. GS conducted the statistical analysis. TY helped in the design of the study. All authors read and approved the final manuscript

## Pre-publication history

The pre-publication history for this paper can be accessed here:


